# Galvanic Anæsthesia

**Published:** 1859-04

**Authors:** 


### *Galvanic Anæsthesia.*

M. Edward Robin lately read a paper before the Academy of Sciences of Paris, wherein he endeavored to show that the diversity of opinion respecting galvanic anæsthesia is owing to its acting either faintly or energetically, and thus producing opposite effects. The author, therefore, contends that the remarkable results obtained in America are not weakened by the failures which have been observed on this side of the Atlantic. Further carefully-conducted experiments are however necessary to settle the amount of confidence which should granted to this kind of an anæsthesia.

We earnestly hope that improvements and adaptations will be discovered in this country to diminish or abolish pain in tooth extraction, seeing the risks accompanying the administration of chloroform have not diminished.—*London Lancet.*

---

### *Statistics on Excision of the Knee-Joint.*

This operation was lately mentioned at a meeting of the Surgical Society of Paris; and doubts respecting its efficacy were raised by Messrs. Marjolin, Robert, and Larrey. Thereupon M. Giraldès set about collecting the cases which have hitherto been published, and presented the following figures:

From 1762 to 1858, the operation was performed 127 times; it proved fatal in 33 cases. The operations may be divided into the following series:

| | | | | |
|---|---|---|---|---|
| 1st series, | from | 1762 to 1830, 19 | operations, | 12 deaths. |
| 2nd " | " | 1830 to 1854, 31 | " | 5 " |
| 3rd " | " | 1854 to 1856, 51 | " | 9 " |
| 4th " | " | 1856 to 1858, 26 | " | 7 " |

Out of the 31 patients of the 2d series, 17 have recovered with a perfect use of the limb.—*Ib.*

---

*Local Anæsthesia.*

M. Claisse proposes, in the *Gazette des Hôpitaux*, to use local anæsthesia. Into a small phial he introduces powdered camphor, so as to occupy one-third of the bottle; and then fills it up with sulphuric ether. With this solution the skin is to be lightly rubbed by means of a sponge fixed to a whalebone; a minute's friction suffices, and teeth may thus be extracted without pain, by applying the lotion to the gums. Other minor painless operations may also thus be performed. When the patient makes difficulties the rubbing should be repeated, as the effect is quickly lost.—*Ib.*

